# Activation of P21-activated protein kinase 2 is an independent prognostic predictor for patients with gastric cancer

**DOI:** 10.1186/1746-1596-9-55

**Published:** 2014-03-12

**Authors:** Chengcheng Gao, Tianheng Ma, Liqun Pang, Rui Xie

**Affiliations:** 1 Department of Gastroenterology, Huai’an First People’s Hospital, Nanjing Medical University, 6 Beijing Road West, Huai’an, Jiangsu 223300, P. R. China

**Keywords:** Gastric cancer, p21-activated kinase 2, Phosphorylation, Immunohistochemistry, Prognosis

## Abstract

**Objective:**

p21-activated kinase (PAK) 2, as a member of the PAK family kinases, is involved in a number of hallmark processes including cell proliferation, survival, mitosis, apoptosis, motility and angiogenesis. However, the clinical significance of the activation of PAK2 in human gastric cancer has not been fully elucidated. The aim of this study was to investigate whether PAK2 expression and its phosphorylation status are correlated with tumor progression and prognosis in gastric cancer.

**Methods:**

Expression patterns and subcellular localizations of PAK2 and Ser20-phosphorylated PAK2 (pSer20PAK2) in 82 gastric cancer patients were detected by immunohistochemistry.

**Results:**

Both PAK2 and pSer20PAK2 immunostainings were localized in the cytoplasm of tumor cells of gastric cancer tissues. Compared with the normal gastric mucosa, the expression levels of PAK2 and pSer20PAK2 proteins were both significantly increased (both P < 0.001). Additionally, the patients displaying the over-expression of PAK2 and pSer20PAK2 proteins were dramatically associated with unfavorable clinicopathologic variables including higher tumor depth (P = 0.022 and 0.036, respectively), greater extent of lymph node metastasis ((P = 0.022 and 0.036, respectively), positive distant metastasis (P = 0.025 and 0.038, respectively) and advanced tumor stage (P = 0.018 and 0.031, respectively). Moreover, the patients overexpressing PAK2 and pSer20PAK2 proteins have poor overall survival rates relative to those without overexpression of these proteins. Furthermore, cox multi-factor analysis showed that PAK2 (p = 0.012) and pSer20PAK2 (p = 0.010) were independent prognosis factors for human gastric cancer.

**Conclusion:**

Our data suggest for the first time that PAK2 activation may be associated with advanced tumor progression and poor prognosis of gastric cancer.

**Virtual slides:**

The virtual slides for this article can be found here: http://www.diagnosticpathology.diagnomx.eu/vs/1236344107120406.

## Introduction

Gastric cancer is one of the most common neoplasms in digestive system with highly malignant and a poor prognosis worldwide, especially in Asia and Africa [1]. It tends to be associated with lymph node metastasis, peritoneal dissemination, and hematogenous metastasis. Despite the advancement of surgical technique and the improvement of anticancer drugs in recent years, gastric cancer is still a leading cause of cancer-related deaths and the 5-year survival rate is approximately 20% [2]. Especially in China, the morbidity of gastric cancer has reached to second with 3,621,000 new cases, whilst the mortality rate ranked third with the proportion of 14.33% annually [3]. It has been demonstrated that the depth of tumor invasion, peritoneal dissemination, hepatic metastasis, and lymph node metastasis are significant factors in determining prognosis [4]. Since tumor invasion and metastasis are very complicated and continuous processes involving multiple steps, regulated at the molecular level by adhesion molecules, protein catabolic enzymes, cellular growth factors, and various angiogenic factors, it is extremely necessary to identify novel and efficient biomarkers for the evaluation of the behavior in tumor development and metastasis in order to predict prognosis and improve therapeutic strategies for patients with gastric cancer.

The p21-activated kinases (PAKs) are a family of serine/threonine protein kinases, which were initially identified as binding partners of the Rho GTPases Cdc42 and Rac1 [5]. The PAK family includes six isoforms (PAK1-6) which play a crucial role in a variety of physiological processes such as motility, survival, mitosis, apoptosis, and hormone signaling [6]. The PAKs are divided into two groups, group I (PAKs 1–3) and group II (PAKs 4–6) based on structural and functional similarities: group I PAKs exist in an inactive homodimer maintained by interactions between the autoinhibitory domain (AID) and kinase domain of PAK monomers; group II PAKs also bind Rac and Cdc42, but they lack an AID, exist as active monomers, and have not been reported to have a scaffolding function [7]. Recent studies have demonstrated that PAKs are overexpressed, hyperactivated or amplified in several human cancers and their role in cell transformation make them attractive therapeutic targets [8]. Especially, PAK2, which has an overall 76% homology with PAK1 and 96% homology in the kinase domain, has a dual role in both cell survival and cell death pathways. It is widely distributed throughout the body and is not only activated by binding with the small G protein complex Cdc42/Rac, but it is also cleaved and activated by caspase-3 and similar proteases [9]. Full length PAK2 is autophosphorylated at eight sites including Ser20, Ser139, Ser141, Ser144, Ser192, Thr402, Thr421 and Thr423, and then activated [9]. Accumulating evidence indicates that PAK2 are either up-regulated or hyperactivated in a variety of human cancers, including ovarian cancer [10] and breast cancer [11]. PAK2 plays an important role in tumor aggressiveness, but its involvement in gastric cancer has not yet clear. The aim of this study was to investigate whether PAK2 expression and its phosphorylation status are correlated with tumor progression and prognosis in gastric cancer.

## Materials and methods

### Patients and tissue samples

Eighty-two patients with gastric cancer (56 males and 26 females), underwent gastrectomy with lymph node dissection between 1992 and 2006 at Department of Gastroenterology were selected in this study. The patients ranged in age from 22 to 88 years (mean 65 years). None of these patients underwent endoscopic mucosal resection, palliative resection, or preoperative chemotherapy, or had synchronous or metachronous multiple cancer in other organs.

Clinicopathological findings were based on the criteria of the tumor node metastasis (TNM) classification of the International Union against Cancer [9]. Histopathological types of gastric cancer were classified into two types, intestinal type and diffuse type. The intestinal type was further classified into three differentiated types: well-differentiated (tub1), moderately differentiated (tub2), and papillary differentiated (pap); and the diffuse type was classified into two undifferentiated types: diffuse-adherent (por1) and diffuse-scattered (tub2).

All patients were followed up after discharge, with X-ray examination and tumor marker assays (carcinoembryonic antigen and carbohydrate antigen 19–9) performed every 1–3 months, computed tomography performed every 3–6 months, and ultrasonography performed every 6 months. Endoscopic examinations were performed when necessary. Postoperative follow-up data were obtained from all patients, with a median follow-up period of 36 months (range 1–138 months).

This study was approved by the Research Ethics Committee of Huai’an No.1 Hospital, Affiliated to Nanjing Medical University, Huai'an, Jiangsu, China. Informed consent was obtained from all of the patients. All specimens were handled and made anonymous according to the ethical and legal standards.

### Immunohistochemistry analysis

Immunohistochemical study for PAK2 and pSer20PAK2 was performed on formalin-fixed, paraffin-embedded, 4-μm-thick tissue sections using the avidin-biotin-peroxidase complex method. In brief, the sections were deparaffinized and dehydrated using a graded series of ethanol solutions. Endogenous peroxidase activity was halted through the administration of 0.3% hydrogen peroxidase and methanol for 20 min. After having been rinsed in phosphate-buffered saline (PBS), the tissue sections were processed in a 0.01 M citrate buffer (pH 6.0) inside a heat-resistant plastic container. Sections were then irradiated in a domestic microwave oven for 20 min. After microwave irradiation, the slides were allowed to cool at room temperature. The following antibodies were applied as the primary antibodies: rabbit polyclonal to PAK2 (#ab45426, Abcam, Cambridge, UK) and rabbit polyclonal to PAK2 (phospho S20) (#ab59359, Abcam, Cambridge, UK). The sections were incubated with the primary antibody overnight at 4°C followed by the secondary antibody. The results were visualized with diaminobenzidine. In each immunohistochemistry run, matched negative controls were stained without primary antibody.

Following a hematoxylin counterstaining, immunostaining was scored by two independent experienced pathologists, who were blinded to the clinicopathological parameters and clinical outcomes of the patients. The scores of the two pathologists were compared and any discrepant scores were treated through re-examining the stainings using a multi-headed microscope with both pathologists simultaneously to achieve a consensus score. Expression of PAK2 and pSer20PAK2 were respectively compared between paired malignant tissues and normal epithelial tissues located distant from the tumor. The number of positive-staining cells showing immunoreactivity on the cytoplasm for both PAK2 and pSer20PAK2 in ten representative microscopic fields was counted and the percentage of positive cells was calculated. The percentage scoring of immunoreactive tumor cells was as follows: 0 (0%), 1 (1-10%), 2 (11-50%) and 3 (>50%). The staining intensity was visually scored and stratified as follows: 0 (negative), 1 (weak), 2 (moderate) and 3 (strong). A final immunoreactive score (IRS) was obtained for each case by multiplying the percentage and the intensity score. The median of the IRS value was used as the cutoff value to divide the patients into high and low expression groups of PAK2 and pSer20PAK2 proteins, respectively.

### Statistical analysis

The software of SPSS version12.0 for Windows (SPSS Inc, IL, USA) and SAS 9.1 (SAS Institute, Cary, NC) was used for statistical analysis. The Kaplan-Meier method was used for survival analysis, and differences in survival were estimated using the log-rank test. Prognostic factors were examined by univariate and multivariate analyses (Cox proportional hazards regression model). Differences were considered statistically significant when p was less than 0.05.

## Results

### Overexpression of PAK2 and pSer20PAK2 in human gastric cancer tissues

**Figure 1:**
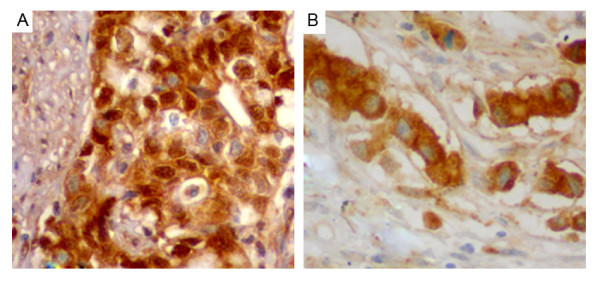
**Immunohistochemical stainings of PAK2 (A) and pSer20PAK2 (B) proteins in the gastric cancer tissues (×400).** Both PAK2 and pSer20PAK2 immunostainings were localized in the cytoplasm of tumor cells of gastic cancer tissues.

Expression patterns and subcellular localizations of PAK2 and pSer20PAK2 in gastric cancer and normal gastric mucosa were detected by immunohistochemistry. As shown in Figure 1, both PAK2 and pSer20PAK2 immunostainings were localized in the cytoplasm of tumor cells of gastric cancer tissues. Compared with the normal gastric mucosa, the expression levels of PAK2 (IRS for gastric cancer vs. normal gastric mucosa: 5.62 ± 1.93 vs. 2.33 ± 0.68, P < 0.001) and pSer20PAK2 (IRS for gastric cancer vs. normal gastric mucosa: 5.99 ± 2.06 vs. 2.27 ± 0.61, P < 0.001) proteins were both significantly increased. Of 82 patients with gastric cancer, 62 (75.61%) and 69 (84.15%) were highly expressed PAK2 and pSer20PAK2, respectively.

### Association of PAK2 and pSer20PAK2 expression with the clinicopathological characteristics of human gastric cancer

Table 1 summarized the association of PAK2 and pSer20PAK2 expression with the clinicopathological characteristics of human gastric cancer. The patients displaying the over-expression of PAK2 and pSer20PAK2 proteins were dramatically associated with unfavorable clinicopathologic variables including higher tumor depth (P = 0.022 and 0.036, respectively), greater extent of lymph node metastasis (P = 0.022 and 0.036, respectively), positive distant metastasis (P = 0.025 and 0.038, respectively) and advanced tumor stage (P = 0.018 and 0.031, respectively).

**Table 1:** Correlations of PAK2 and pSer20PAK2 expression with the clinicopathological features of primary gastric cancer

**Features**	**No. of cases**	**PAK2 expression**		**P**	**pSer20PAK2**		**P**
		**High (%)**	**Low (%)**		**High (%)**	**Low (%)**	
**Age (years)**	82	65.32 ± 11.06	64.59 ± 11.88	NS	65.17 ± 11.29	64.99 ± 11.81	NS
**Gender**							
Male	56	42 (75.00)	14 (25.00)	NS	47 (83.93)	9 (16.07)	NS
Female	26	20 (76.92)	6 (23.08)		22 (84.62)	4 (15.38)	
**Histopathological type**							
* Intestina type*							
pap	3	2 (66.67)	1 (33.33)	NS	2 (66.67)	1 (33.33)	NS
tub1	19	15 (78.95)	4 (21.05)		16 (84.21)	3 (15.79)	
tub2	22	16 (72.73)	6 (27.27)		18 (81.82)	4 (18.18)	
*Diffuse type*							
por1	12	8 (66.67)	4 (33.33)	NS	10 (83.33)	2 (16.67)	NS
por2	26	21 (80.77)	5 (19.23)		23 (88.46)	3 (11.54)	
**Tumor depth (pT)**							
pT1 ~ pT2	62	42 (67.74)	20 (32.26)	0.022	49 (79.03)	13 (20.97)	0.036
pT3 ~ pT4	20	20 (100.00)	0 (0)		20 (100.00)	0 (0)	
**Lymph node metastasis (pN)**							
pN0 ~ pN1	66	46 (69.70)	20 (30.30)	0.022	53 (80.30)	13 (19.70)	0.036
pN2 ~ pN3	16	16 (100.00)	0 (0)		16 (100.00)	0 (0)	
**Distant metastasis (pM)**							
pM0	76	56 (73.68)	20 (26.32)	0.025	63 (82.89)	13 (17.11)	0.038
pM1	6	6 (100.00)	0 (0)		6 (100.00)	0 (0)	
**pStage**							
I ~ II	59	39 (66.10)	20 (33.90)	0.018	46 (77.97)	13 (33.90)	0.031
III ~ IV	23	23 (100.00)	0 (0)		23 (100.00)	0 (0)	
**Lymphatic invasion**							
Negative	35	26 (74.29)	9 (25.71)	NS	29 (82.86)	6 (17.14)	NS
Positive	47	36 (74.60)	11 (25.40)		40 (85.11)	7 (14.89)	
**Venous invasion**							
Negative	58	42 (72.41)	16 (17.59)	NS	49 (84.48)	9 (15.52)	NS
Positive	24	20 (83.33)	4 (16.67)		20 (83.33)	4 (16.67)	
**Hematogenous recurrence**							
Negative	72	55 (76.39)	17 (23.61)	NS	61 (84.72)	11 (15.28)	NS
Positive	10	7 (70.00)	3 (30.00)		8 (80.00)	2 (20.00)	
**Peritoneal recurrence**							
Negative	72	55 (76.39)	17 (23.61)	NS	61 (84.72)	11 (15.28)	NS
Positive	10	7 (70.00)	3 (30.00)		8 (80.00)	2 (20.00)	

Note: ‘NS’ refers to ‘no significant’.

### Overexpression of PAK2 and pSer20PAK2associate with unfavorable prognosis in human gastric cancer

**Figure 2:**
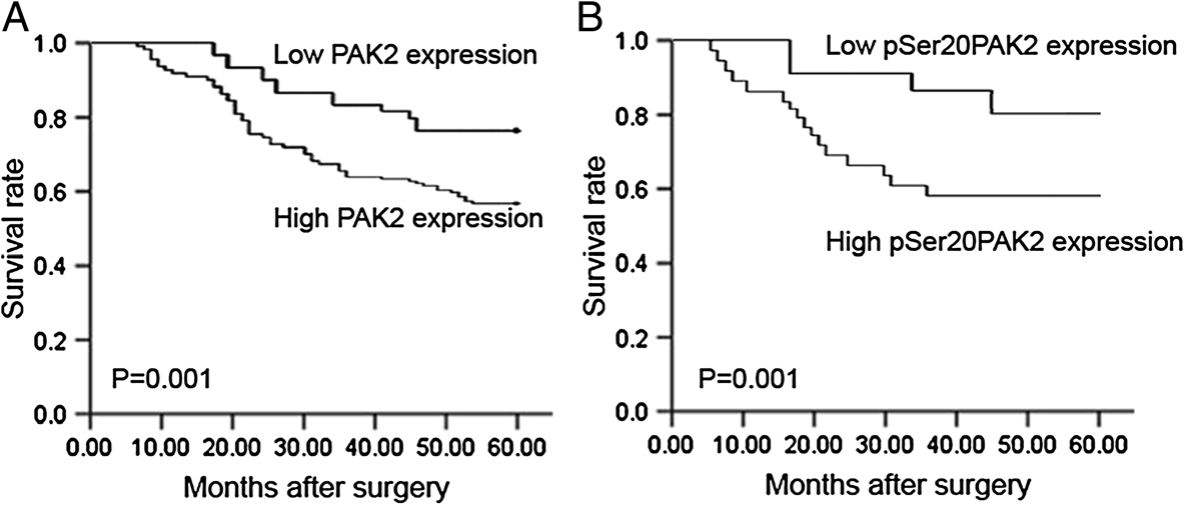
**Postoperative 5-year survival curves of patients according to the expression patterns of PAK2 (A) and pSer20PAK2 (B) proteins.** Statistical analysis showed that the 5-year survival in patients with high PAK2 and pSer20PAK2 expressions were both significantly shorter than those with low expressions (both P = 0.001).

No patient died of postoperative complications within 30 days at the beginning of the study period. The 5-year survival rates of patients with high PAK2 and pSer20PAK2 expression were respectively 58.01% and 57.97%, whereas the rates for patients with low PAK2 and pSer20PAK2 expression were respectively 79.03% and 79.71%. Statistical analysis showed that the 5-year survival in patients with high PAK2 and pSer20PAK2 expressions were both significantly shorter than those with low expressions (both P = 0.001; Figure 2).

Table 2 shows the univariate and multivariate analyses of factors related to patient prognosis. The univariate analysis showed that the following factors were significantly related to postoperative survival: depth of tumor invasion (P < 0.001), lymph node metastasis (P < 0.001), venous invasion (P < 0.001), PAK2 expression (P = 0.001) and pSer20PAK2 expressions (P = 0.001). Multivariate regression analysis indicated that PAK2 expression (P = 0.01), pSer20PAK2 expressions (P = 0.01), depth of invasion (P = 0.002) and lymph node metastasis (P < 0.001) were independent prognostic factors.

**Table 2:** Univariate and multivariate analyses of prognostic factors in gastric cancer

**Independent factors**	**Univariate P**	**Multivariate P**	**Hazard ratio**	**95 % confidence interval**
**Tumor depth (pT)**				
pT1 and pT2/pT3 and pT4	<0.001	0.002	2.821	1.312 ~ 6.263
**Lymph node metastasis (pN)**				
Negative/positive	<0.001	<0.001	7.292	2.575 ~ 26.327
**Venous invasion**				
Negative/positive	<0.001	NS	1.673	0.910 ~ 4.122
**PAK2 expression**				
Low/high	0.001	0.012	3.681	1.274 ~ 11.066
**pSer20PAK2 expression**				
Low/high	0.001	0.012	3.624	1.196 ~ 11.228

Note: ‘NS’ refers to ‘no significant’.

## Discussion

Gastric cancer patients with the same tumor stage present different clinical courses and have different prognosis. This heterogeneity is present at the molecular level and has a genetic predisposition to it. An increasing number of molecular markers identified by several research groups to diagnose patients with gastric cancer at an early stage and to screen the patients with a high risk of developing recurrence or metastases from those patients with a low risk [12-17]. However, the fundamental molecular mechanism underlying tumorigenesis and tumor progression in gastric cancer is poorly understood and has not been fully elucidated. Thus, it is of great significance to identify effective indicators to better understand the biological basis for the survival of gastric cancer patients in order to provide important clinically relevant insights into disease management. In the current study, we demonstrated that the expression levels of PAK2 and pSer20PAK2 proteins were significantly higher in gastric cancer than in normal gastric mucosa. There were significant associations between PAK2, pSer20PAK2 expression and aggressive clinicopathological features. The overexpression of PAK2 and pSer20PAK2 proteins were both identified as independent prognostic factors in gastric cancer. These findings suggest that the activation of PAK2 may be a valuable diagnostic marker and therapeutic target for gastric cancer. To the best of our knowledge, this is the first report to determine the correlation between PAK2, pSer20PAK2 expression and clinical factors in gastric cancer.

The primary hallmark of cancer is the growth of tumor cells and the ability to form tumors [18]. PAKs were originally shown to be important for transformation in experiments where a kinase dead mutant of PAK was expressed in fibroblasts together with an oncogenic Ras mutant [19]. Subsequently, it has been demonstrated that PAKs are involved in stimulation of cell proliferation (including anchorage-independent growth), stimulation of cell survival (inhibition of apoptosis), and stimulation of cell motility, which are all the most prominent hallmarks of cancer [19]. Each of these three hallmarks has at least one known target in a well-established signaling pathway, which is a direct PAK target. In the PAK family, PAK2 is unique because it is not only activated by binding with the small G protein complex Cdc42/Rac, but it is also cleaved and activated by caspase-3 and similar proteases. After binding to Cdc42/Rac, the autoinhibitory activity of PAK2 is attenuated, leading to the autophosphorylation and activation of PAK2 [20-22]. Recent studies have established the function of PAK2 in cancer and identified it as an invasiveness-associated gene which is implicated with cancer proliferation and survival. For example, Li et al. [11] revealed that PAK2 was highly expressed in breast cancer cell lines and breast invasive ductal carcinoma tissue suggesting that highly expressed PAK2 might promote breast cancer progression and restrain the cell death response induced by chemotherapeutic drugs; Siu et al. [10] indicated that PAk2 and pPAK2 were both overexpressed in ovarian cancer cell lines and clinical samples of ovarian cancers compared with normal cell lines and benign ovarian lesions/inclusion cysts. They also found that knockdown of PAK2 in ovarian cancer cell lines reduced cell migration and invasion but did not affect cell proliferation and apoptosis, suggesting that PAK2 and pPAK2 may play important roles in ovarian carcinogenesis. In line with these previous findings, our data showed the upregulation of PAK2 and pSer20PAK2 in gastric cancer tissues, implying the activation of PAK2 in tumorigenesis of this disease. Then, our statistical analysis found that the overexpression of PAK2 and pSer20PAK2 proteins may be associated with higher tumor depth, greater extent of lymph node metastasis, positive distant metastasis and advanced tumor stage, suggesting that the activation of PAK2 may be involved in the aggressive tumor progression of gastric cancer. More interestingly, the aberrant expressions of PAK2 and pSer20PAK2 proteins were both independent prognostic factors for gastric cancer.

## Conclusion

In conclusion, our data suggest for the first time that the activation of PAK2 may be associated with advanced tumor progression of gastric cancer. The overexpression of PAK2 and pSer20PAK2 proteins may be recognized as independent prognostic markers for overall survival, therefore, the detection of these proteins may be helpful for predicting clinical outcome for patients with gastric cancer.

## Competing interests

The authors declare that they have no competing interests.

## Authors’ contributions

GCC carried out the experimental studies and drafted the manuscript. PLQ and MTH carried out part of the experimental studies. XR designed the experiments and modified the manuscript. All authors read and approved the final manuscript.
